# Simultaneous miRNA and mRNA Transcriptome Profiling of Differentiating Equine Satellite Cells Treated with Gamma-Oryzanol and Exposed to Hydrogen Peroxide

**DOI:** 10.3390/nu10121871

**Published:** 2018-12-02

**Authors:** Karolina A. Chodkowska, Anna Ciecierska, Kinga Majchrzak, Piotr Ostaszewski, Tomasz Sadkowski

**Affiliations:** Department of Physiological Sciences, Faculty of Veterinary Medicine, Warsaw University of Life Sciences-SGGW, Nowoursynowska 159, 02-776 Warsaw, Poland; landadelka82@o2.pl (K.A.C.); anna_ciecierska@sggw.pl (A.C.); kinga_majchrzak@sggw.pl (K.M.); piotr_ostaszewski@sggw.pl (P.O.)

**Keywords:** gamma-oryzanol, equine satellite cells, miRNA, target genes, hydrogen peroxide, muscle injuries, apoptosis, oxidative stress

## Abstract

Gamma-oryzanol (GO) is a popular supplement for performance horses, dogs, and humans. Previous studies indicated that GO supplementation decreases creatine kinase activity and lactate level after exercise and may affect oxidative stress in Thoroughbred horses. GO may change genes expression in equine satellite cells (ESC). The purpose of this study was to evaluate the effect of GO on miRNA, gene expression, oxidative stress, and cell damage and viability in differentiating ESC pretreated with hydrogen peroxide (H_2_O_2_). ESCs were obtained from a young horse’s skeletal muscle. ESCs were pre-incubated with GO (24 h) and then exposed to H_2_O_2_ for one hour. For the microRNA and gene expression assessment, the microarray technique was used. Identified miRNAs and genes were validated using real time-quantitative polymerase chain reaction. Several tests related to cell viability, cell damage, and oxidative stress were performed. The microarray analysis revealed differences in 17 miRNAs and 202 genes between GO-treated and control ESC. The tests related to apoptosis, cell viability, and oxidative stress showed that GO affects these processes to varying degrees. Our results suggest that GO can change miRNA and gene expression and may impact the processes involved in tissue repairing after an injury.

## 1. Introduction

Gamma-oryzanol (GO), a component of rice bran, is a mixture of ferulic acid esters, which are formed by esterification of the hydroxyl group of sterols (campesterol, stigmasterol, and *β*-sitosterol) or triterpene alcohols (cycloartanol, cycloartenol, 24-methylenecycloartanol, and cyclobranol) with the carboxylic acid group of ferulic acid [1,2,3]. Previous studies suggested that GO has a variety of biological effects, including cholesterol-lowering, glucose metabolism [4,5,6], anti-inflammatory [7], and anti-oxidant activities [6]. In vitro GO antioxidant activity was reported to be four times higher than vitamin E [8] and has been used to treat hyperlipidemia and increase muscle mass [9]. Studies have also shown that GO increases not only improved muscle strength due to resistance training [10] but also the concentration of growth hormone, testosterone, and other anabolic or muscle building hormones. GO is known to affect gene expression and several metabolic pathways related to inflammation [11,12] and oxidative stress [13]. Szczesniak et al. [14] demonstrated that GO may affect different processes in differentiating equine satellite cells (ESCs), including inhibition of myoblast differentiation, and increased proliferation and differentiation.

Muscle wasting in horses increases the risk of morbidity/mortality in primary muscle diseases and secondary muscle disorders. A few types of equine muscle disorders and myopathies cause the exercise intolerance [15]. Physical exercise can cause oxidative stress. Several studies showed that the pathogenesis of exercise-induced myopathies and inflammation in horses may be related the reactive oxidative species (ROS) generated during different kinds of effort [16,17]. ROS may damage not only the basic cellular structure but may also cause DNA damage [18]. 

Apoptosis, which is a type of cell death occurring during the development of mammalian muscles, plays an important role in removing unnecessary, damaged, or potentially dangerous cells [19]. A number of factors induce apoptosis, like DNA damaging factors, oxidative stress, or partial ischemia. As mentioned above, effort and physical exercise may lead to disturbance and damage of skeletal muscle. It has also been demonstrated that intense exercise could lead to apoptosis in skeletal muscles [20]. The mechanism in which post-exercise apoptosis is activated may be related to oxidative stress and the generation of free radicals. Satellite cells, which play a fundamental role in muscle regeneration, are sensitive to apoptotic cell death as they proliferate [21] and differentiate [22]. The first 24 h after myotrauma is a critical period during muscle regeneration. During this stage, several molecular changes prior to cell division occur. However, the sensitivity of satellite cells to apoptotic death within this time has not been determined. In healthy organisms, antioxidants protect satellite cells against ROS. However, excessive ROS production (which is observed during exercise, some myopathies, and muscle disease) signals the potential vulnerability of satellite cells to apoptosis following myotrauma. Apoptosis may be a mechanism responsible for the depletion of satellite cells in old animals and degenerative skeletal muscle [21,23].

A number of genes and miRNAs were previously described as being involved in cellular and metabolic processes, observed during effort, inflammation, or aging in muscular tissue [24,25]. We hypothesize that GO pre-incubation may change miRNA and the gene profile of differentiating equine satellite cells treated with hydrogen peroxide (H_2_O_2_). Our observations suggest that GO may affect various physiological and pathological processes in muscular cells in multiple ways. Our study may provide a supplement to existing knowledge on the effects of gamma-oryzanol on muscle cells. The results can be a source of information for further research on the effects of gamma-oryzanol on muscle cell regeneration after damage associated with exercise and certain diseases (myopathies), oxidative stress, or the apoptosis-related effects of this compound.

## 2. Material and Methods

### 2.1. Muscle Samples and Cell Culture

Samples were obtained from an equine slaughter house. Muscle cuts were taken from six 6-month old stallions. All the horses were previously clinically examined. Muscle samples were washed in 100 mL phosphate buffered saline (PBS, Invitrogen, Carlsbad, USA) mixed with *Penicilinum crystalicum* (20,000/40,000 units (U), PC; Polfa, Tarchomin, Poland). Connective and fat tissue were separated from the sample, which was suspended in mixture of fetal bovine serum (FBS, Thermo Fisher Scientific, Waltham, MA, USA) and 10% dimethyl sulfoxide (DMSO, Sigma Aldrich, Poznań, Poland). After gradually freezing to −80 °C, tissue was snap-frozen in liquid nitrogen and stored until use.

### 2.2. Satellite Cells Isolation

Equine satellite cells (ESC) were isolated based on the same protocol as described by Chodkowska et al. [25]. The growth medium was changed every two days. Based on the cell viability (MTT; 3-(4,5-dimethylthiazol-2-yl)-2,5-diphenyltetrazolium bromide assay) and fusion index, the best primary cell line was chosen [25,26].

### 2.3. Satellite Cells Proliferation, Differentiation, and Treatment

**Figure 1:**
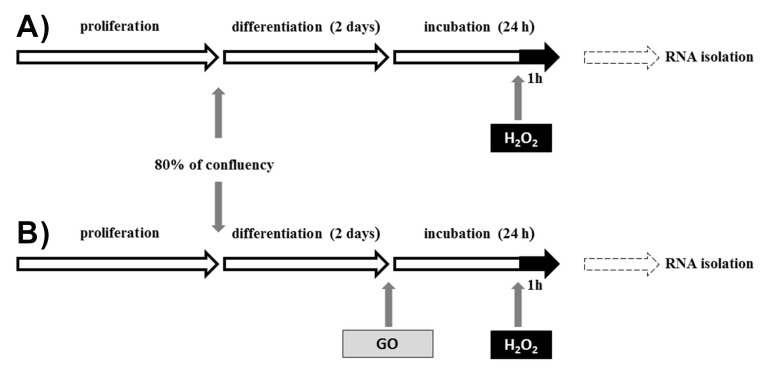
Experimental design: (**A**) control group (ESCs incubated with hydrogen peroxide) and (**B**) GO-treated group incubated with hydrogen peroxide.

On the 10th day of proliferation, cells were trypsinized. The next step was counting the cells using a Scepter Cell Counter (Merck Millipore, Darmstadt, Germany) and transferring 30,000 cells/well to Cellware 6-well plate covered by Collagen I (Greiner Bio-One, Monroe, NC, USA). When the cells obtained 80% confluence, the proliferation medium was replaced by differentiation medium (2% HS/ Dulbecco’s Modified Eagle Medium (DMEM)/AB). After the 2nd day of differentiation, 0.125 µM of GO (TCI Chemicals, Portland, USA), dissolved in 0.04 µL/mL DMSO as a vehicle, which was also used in the control medium, was added for 24 h. The MTT assay was used to choose the concentration of GO and H_2_O_2_ [25]. During the last part of the experiment (the last one hour of GO incubation), H_2_O_2_ (3 mM, Sigma Aldrich, Poznań, Poland) was added. The main purpose of H_2_O_2_ administration was to cause cell damage (Figure 1).

### 2.4. RNA Isolation 

After the GO/H_2_O_2_ incubation, differentiated ESCs were scraped for the total RNA isolation (*n* = 6 each from GO-treated and control groups) using a miRNeasy Mini Kit (Qiagen, Hilden, Germany) according to the manufacturer’s protocol previously described by Chodkowska et al. [25]. Only RNA samples with RNA integrity number (RIN) number ≥ 9.2 were included in the further analysis. 

### 2.5. Microarray Analysis

Custom-made equine miRNA 8 × 15 K microarray slides by Agilent Technologies (Santa Clara, CA, USA), designed using Agilent eArray platform; GEO database: GPL20990, were used for miRNA profiling. 

MiRNA was isolated from 8 equine satellite cell cultures for both GO-pre-treated (*n* = 8) and the control group (*n* = 8). The isolation procedure was previously described by Chodkowska et al. [25]. In this procedure, 100 ng total RNA of each sample was used for further analysis using a commercial labeling and hybridization kit according to manufacturer protocol (Hyb Kit (Version 2.3, December 2010, Agilent Technologies, Santa Clara, USA). Slides were scanned with a Microarray Scanner (model G2565CA) with SureScan High-Resolution Technology (Agilent Technologies, Santa Clara, CA, USA).

All extracted data were normalized using the standard procedures described in the Agilent Feature Extraction (FE) Software Version 10.7.3.1 (Agilent Technologies, Santa Clara, CA, USA). 

The Horse Gene Expression Microarray, 4 × 44 K (*n* = 4) (Agilent Technologies, Santa Clara, CA, USA) was used to analyze gene expression profile. We used 825 ng of cDNA isolated from the GO-treated and the control group. The whole procedure was previously described by Chodkowska et al. [25].

The data were statistically analyzed using Gene Spring 13.0 software (Agilent Technologies, Santa Clara, CA, USA). The statistical significance of the differences was evaluated using Student’s test (*p* < 0.05). A Benjamini and Hochberg multiple testing correction was performed. False Discovery Rate (FDR ≤ 0.05) and fold change (FC) ≥ 1.3 were considered as statistically significant. Microarray data were deposited at the Gene Expression Omnibus data repository under the number for miRNA GSE122580 and for cDNA GSE122098.

### 2.6. Real-Time qPCR

MicroRNAs and genes involved in muscle injuries and tissue regeneration, muscle development, cellular proliferation, differentiation, migration, apoptosis, lipid and protein metabolism, and oxidative stress were selected for real-time quantitative polymerase chain reaction (RT-qPCR) validation and further analysis.

For miRNA RT-qPCR validation, miRCURY LNA^TM^ Universal RT microRNA PCR kit (Exiqon, Vedbaek, Denmark) was used based on the same protocol presented by Chodkowska et al. [25]. 

Primers were chosen based on the miRNA sequences assigned to microarray probes and provided by Exiqon (Vedbaek, Denmark). The relative gene and miRNA expression were calculated according to the following formula: ΔΔ Ct method (ΔΔCT = ΔCT (sample) − ΔCT (control)) using GenEX 6 software (MultiD, Göteborg, Sweden). 

Statistical analysis was performed using 2-tailed Student’s test (*t*-test). Values of *p* < 0.05 were considered statistically significant. U6 snRNA reference was used based on previous studies and the manufacturer recommendation (Exiqon, Vedbaek, Denmark). The primers used in the assay are shown in Table 1A.

To verify microarray results for gene expression profiling, RT-qPCR was applied. All the steps for the RT-qPCR procedure were determined based on the protocols previously described by Chodkowska et al. [25]. 

The sequences of validated genes were obtained from Ensembl or the NCBI Gene database. Table 1b shows the primers used in this assay. All the primers were designed using Primer-BLAST NCBI, M.Zucker m-fold Web Server for nucleic acid folding and hybridization prediction. As a reference gene, *gapdh* was used. All the primers were prepared by Oligo IBB (Polish Academy of Science, Warsaw, Poland).

### 2.7. Ontological Analysis and Target Gene Prediction

Pathway Studio Web Mammal (Elsevier, Amsterdam, Netherlands) was used to analyse in detail the role of GO-modulated miRNAs and genes in various metabolic and signal pathways. Relationships between all differentially expressed miRNAs and genes were visualized with Pathway Studio’s Build Pathway functionality, which is based on the wave-propagation algorithm developed for the navigation through complex networks. Find Direct Links/All Objects Directions Algorithm was used in this analysis. 

To predict microRNA target genes, the TargetScan database was used. All identified GO-modulated miRNAs were analysed. Predicted targets of each miRNA family were sorted by total context+ score. All conserved/non-conserved miRNA families and target sites were scanned [25].

Ontological analyses revealing molecular functions, biological processes and pathways of miRNA targets were calculated in Database for Annotation, Visualization and Integrated Discovery (DAVID 6.7) online tool by Fisher’s exact test with *p* ≤ 0.05 [25].

### 2.8. Cell Viability, Cell Damage, and Oxidative Stress

Tests related to the oxidative stress, cell viability, and cell damage were performed to assess the impact of GO on the processes occurring in the cells after incubation with H_2_O_2_. Experimental conditions (incubation time, doses of GO and H_2_O_2_) were the same as in the procedure related to the microarray and RT-qPCR analysis.

All the commercial tests: CellROX^®^ Green Reagent Kit (containing SYTOX Red Dead Cell test; Life Technologies, Carlsbad, CA, USA), Total Antioxidant Capacity (TAC) Assay Kit (Abcam, San Francisco, CA, USA), Lipid peroxidation Assay Kit (Sigma Aldrich, Poznań, Poland), and JC-1 (Sigma Aldrich, Poznań, Poland) that were used to measure oxidative stress, cell death, lipid peroxidation, and mitochondrial depolarization in ESC, respectively, were performed based on the manufacturer’s protocol, previously described by Chodkowska et al. [25].

All acquired data were analysed using GraphPad software (GraphPad Software, La Jolla, CA, USA) and FlowJo (TreeStar, Ashland, OR, USA) (for qualitative flow cytometry assays).

## 3. Results

### 3.1. MiRNA Expression and Gene Ontology 

Analysis of the expression of differentiating equine satellite cells pre-incubated with GO (24 h) and exposed to H_2_O_2_ (1 h) revealed differences in the expression of 17 miRNAs. Among them, 5 demonstrated higher expression and 12 lower expression when compared to control (no GO pre-incubation) (Table 2). 

Identified miRNAs were divided into several groups according to previously described function: myomiRs and miRNA related to muscle tissue (miR-133a, miR-222, miR-204, miR-208b), cell proliferation and differentiation (miR-10a, miR-133, miR-208b, miR-222, miR-675, miR-708), apoptosis (miR-133a, miR-222), lipid and protein metabolism (miR-133a, miR-29c), tissue injury, muscle diseases, immunity response, and inflammation (also muscle dystrophies) regeneration (miR-142-3p, miR-199b, miR-222, miR-675 5), and muscle hypertrophy (miR-199b, miR-212). 

### 3.2. mRNA Expression and Gene Ontology

The difference in the expression of 202 genes (FC > 1.0) was analyzed using a cDNA equine microarray. After removal of the double or unmapped genes, we identified 161 genes that have been identified in different animal species. From this group, 148 genes were previously identified in *Equus caballus* (Table S1).

Among the identified genes, a large number were related to metabolic processes (primary metabolic processes such as protein, lipid, nucleobase-containing compound, and carbohydrate metabolic processes), and cellular processes like cell communication, cell cycle, and cytokinesis. Several genes are known to be involved in a process related to myogenesis, immunity, and apoptosis.

### 3.3. RT-qPCR Validation

Based on the identified miRNA and gene relations with processes—such as muscle development, proliferation, differentiation, hypertrophy, regeneration, inflammation, and oxidative stress—and ontological analysis using DAVID and Pathway Studio, three miRNAs (miR-133a, miR-345, miR-675) were selected as a single representative of the processes for further RT-qPCR analysis.

The analysis confirmed statistically significant differences in their expression between GO and control conditions (Table 3). Two validated miRNAs showed the same trend as microarray results. One, miR-675, presented a different trend from the microarray results.

Based on the same criteria as for miRNA, we chose five genes related to muscle development, oxidative stress, cell proliferation, differentiation, hypertrophy, and tissue regeneration for RT-qPCR validation. Four of them were statistically significant and presented the same trend as in the microarray results.

### 3.4. Prediction of miRNA Targets and Gene Ontology Enrichment

The entire set of 17 modulated miRNAs was analysed in order to identify their gene targets using TargetScan. This analysis revealed 2363 unique targets (Table S2). The highest potential in the regulation of target genes was observed in miRNAs: miR-133a and miR-142, which were shown to affect over 300 targets. Lower potential was observed in miR-29c and miR-675, for which the number of target genes ranged from one up to 10. All identified genes from the GE microarray and all predicted target genes for identified miRNAs from TargetScan analysis were compared in order to select genes regulated by GO-pre-incubation in ESC cultures exposed to H_2_O_2_. As a result of this analysis, we determined 22 potential differentially expressed target genes (DET) for the identified miRNAs.

**Figure 2:**
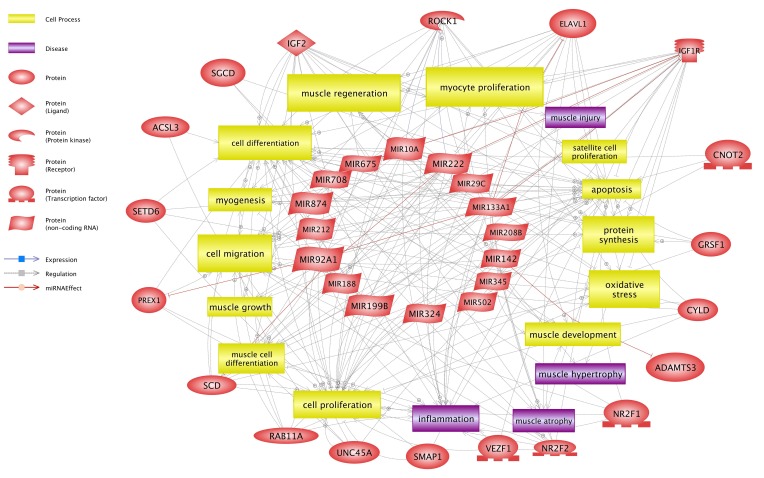
miRNAs and their differentially expressed target genes (DET) in combination with processes that may be associated with muscle tissue during injury and regeneration.

Ontological analysis showed that targets of the identified miRNAs were significantly associated with cellular processes, muscle organ development, proteolysis involved in cellular protein catabolic process, muscle cell differentiation, positive regulation of biological processes, protein catabolic process, proteolysis, cell death, apoptosis, regulation of cell proliferation, and positive regulation of inflammatory process (Figure 2).

Signaling pathway analysis showed that 17 identified miRNAs could influence DET involved in several important pathways related to the physiological and pathological damage to muscle tissue and related GO activity.

### 3.5. Cell Viability, Cell Damage, and Oxidative Stress

#### 3.5.1. Cell Viability

To measure cell viability, the SYTOX Red Dead Cell test was used (as a component of CellROX Green Reagent kit). In this test an increased cell viability and decreased amount of dead cells were observed in ESC cells pre-treated with GO and incubated with H_2_O_2_, and then in a control group (incubated only with H_2_O_2_).

**Figure 3:**
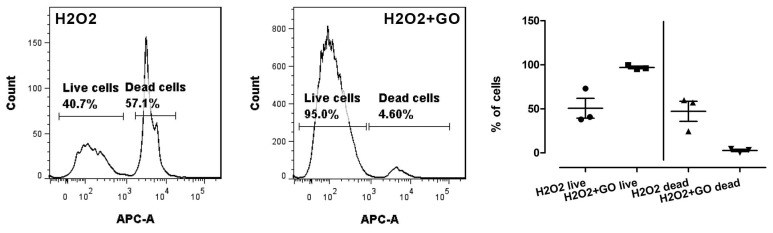
Effect of GO on cell viability measured by SYTOX Red Cell Dead assay. Statistical analysis was performed using the one-way ANOVA (*p* < 0.05). H_2_O_2_ denotes cells without GO treatment, exposed to H_2_O_2_; H_2_O_2_+GO denotes cells treated with GO and exposed to H_2_O_2._

All the results from this test were statistically significant. The results of the SYTOX Red Dead Cell are presented on a Figure 3.

#### 3.5.2. Oxidative Stress and Cell Damage

**Figure 4:**
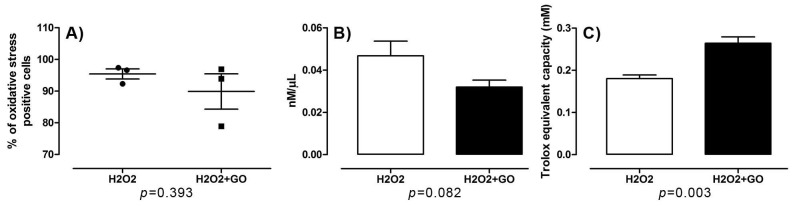
Effect of GO on (**A**) oxidative stress, (**B**) lipid peroxidation, and (**C**) total antioxidant capacity in equine satellite cell cultures exposed to hydrogen peroxide. (**A**) Each value is the mean ± standard error of the results from three different plates (*n* = 3). Statistical analysis was performed using the unpaired *t*-test (two tailed) (*p* < 0.05); (**B**) Each value is the mean ± standard error (*n* = 6). Statistical analysis was performed using the unpaired *t*-test (two tailed) (*p* < 0.05); (**C**) Data are expressed as means ± standard (*n* = 6). Statistical analysis was performed using the unpaired *t*-test (two tailed) (*p* < 0.05).

Oxidative stress was measured using CellROX^®^ Green Reagent. Surprisingly, there was no significant difference between groups (Figure 4a). Similar results were obtained using the test for lipid peroxidation. There were no statistically significant differences between the GO-pre-treated group and control. However, a higher lipid peroxidation trend was observed in a control group. This, in turn, may indicate that GO has antioxidant activity and protects against free radicals. (Figure 4b).

Results obtained from a Total Antioxidant Capacity (TAC) assay showed significant differences between GO-pre-treated and control group. Higher antioxidant capacity was observed in GO-pre-treated group (Figure 4c).

**Figure 5:**
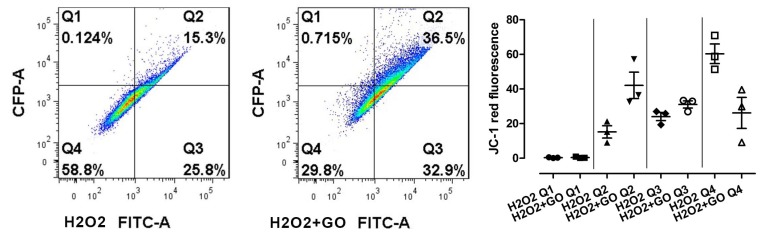
Qualitative flow cytometry assay for mitochondrial depolarization. Data are expressed as mean ± standard error (*n* = 3). Statistical analysis was performed using the one-way ANOVA (*p* < 0.05). H_2_O_2_ denotes cells without GO treatment, exposed to H_2_O_2_; H_2_O_2_+GO denotes cells treated with GO and exposed to H_2_O_2._; Q1 population: % of aggregates; Q2 population: % of monomers + aggregates; Q3 population: % of monomers; Q4 population: % of monomers (−)/aggregates (−).

A qualitative flow cytometry assay for mitochondrial depolarization (JC-1) showed significant differences between the Q2 population (monomers + aggregates in %) and Q4 population (JC-1) in GO-pre-treated groups and control. There was no significant difference between Q1 population (% of aggregates) and Q3 population (% of monomers) (Figure 5).

## 4. Discussion

### 4.1. Gamma-Oryzanol Affects miRNAs and Genes Related to Muscle Cell Proliferation and Differentiation and Processes Related to Muscle Cell Injury and Regeneration

MicroRNAs and their role in muscular tissue are being intensively studied, especially in humans and animal models associated with different kinds of dystrophies, effort, and injuries [27]. Knowledge is still limited concerning their function in injured equine muscle, influence on organ development, and their reaction to dietary factors like commonly used supplements in equine sport and veterinary practice. In this study, **s**imultaneous miRNA and mRNA transcriptome profiling of differentiating equine satellite cells treated with gamma-oryzanol and exposed to hydrogen peroxide was investigated. Microarray analysis showed differences in the expression of 17 miRNAs and 161 genes. Among the identified miRNAs and genes, several appear to be interesting due to the previously described function and their relation to the action of gamma-oryzanol.

### 4.2. Proliferation, Differentiation, and Muscle-Related miRNA and Genes

Among identified miRNAs, a large group was previously described as related to muscle development, myogenesis, and muscle cell proliferation and differentiation. miR-133a, known as myomiR, has distinct roles in modulating skeletal muscle proliferation and differentiation in cultured myoblasts in vitro [28,29]. The second miRNA from this group, miR-222, modulates differentiation and maturation of skeletal muscle cells [30,31], promotes skeletal muscle regeneration in satellite cells [32], and is related to oxidative stress in various tissues [32,33]. The newest studies suggest that miR-29c also participates in muscle development and is closely related to muscle cell proliferation and differentiation [34]. Another identified miRNA, miR-10a, plays essential roles in cardiogenesis and is associated with cardiomyocyte proliferation and differentiation [35]. miR-208b is known as a diagnostic marker of cardiac muscle injury that reflects myocardial damage [36]. Another miRNA, miR-675, induces myogenesis and differentiation of satellite cells during regeneration and becomes a diagnostic marker for muscle injury [37] and cardiomyocytes apoptosis [38]. Notably, miR-133 and miR-208 are up-regulated and miR-324 is down-regulated [39] (the same expression trend was observed in GO-treated group) in most of the exercise regimens, indicating their role in hypertrophy. Moreover, we showed that delivery of miR-133a (local injection) into skeletal muscle promoted muscle regeneration and prevented scar formation [40], and a local injection of miR-1, miR-133, and miR-206 accelerates muscle morphology and functional regeneration. Another miRNA, miR-502-5p, was previously described in cellular models of differentiation of satellite cells into myoblasts and myotubules [41]. However, the authors observed that this miRNA was up-regulated in normal muscle tissue processes. Surprisingly, in our study, we observed decreased expression of miR-502 in an experimental group. Other identified miRNAs, miR-199, miR-142-3p, and miR-212, play important roles in different kinds of cardiac hypertrophy [42]. However, their role in skeletal muscle hypertrophy is still unknown.

Several miRNAs that were identified in our experiment are known to regulate proliferation and differentiation in various kinds of tissues: miR-188 osteoblast and adipocyte [43], and suppress autophagy and myocardial infarction [44], but may also be involved in different physiological and pathological processes in skeletal muscle tissue. Barrey et al. [45] observed differences in miR-188 expression in different horse breeds (French trotter vs. Norman cob). MiR-142-3p balances proliferation and differentiation of mesenchymal cells [46] and affects cardiac and muscular cell fate [47]. Dmitriev et al. [48] observed miR-345 down-regulation in facioscapulohumeral muscular dystrophy; however, there are not much data about this miRNA in other physiological and pathological processes in skeletal muscle. Another miRNA, miR-874, regulates myocardial necrosis [49], and knockdown of this miRNA attenuated necrosis in the cellular model. MiR-708 overexpression results in increased cell proliferation, migration, and invasion [50].

Several identified genes were previously described as being related to various pathological and physiological processes in muscle and other various tissues. Among this group, *nf2r2* (also known as *coup-tfII*) presented the highest overexpression in the experimental group (GO-treated). This is a member of the nuclear orphan receptor superfamily and is broadly detected in stem/progenitor cells in different kinds of tissues and has a profound impact on adult stem cell biology. Xie et al. [51,52] observed that overexpression of *nf2r2* was related to muscle dystrophies and is crucial for vertebrate myogenesis, with particular emphasis on the skeletal and cardiac muscles. Lee et al. [53] noticed COUP-TFII protein expression level is high in undifferentiated progenitors and gradually declines during differentiation. The authors also observed that, similarly, COUP-TFII mRNA reduces as early as 12 h post-differentiation and continuously drops until day four—the time for cultured myotube maturation. Perhaps the situation that we observed in the GO-treated group is related to a level of cell damage in ESCs after H_2_O_2_ incubation.

Among the minority of transcripts that are up-regulated are genes with known roles in myogenesis, including *elavl1*, which encodes HuR, a myogenic differentiation antigen 1 (MyoD) mRNA-stabilizing protein [54], consistent with the induction of MyoD. This induction occurs as early as three hours following isolation of myofiber-associated satellite cells [55]. One of the best-characterized is HuR (Hu antigen R; ELAVL1). HuR stabilizes different mRNAs by binding to Au-rich elements found in 3′UTR and it is implicated in various physiological and pathological processes such as cell growth, differentiation, and inflammation. *elavl1* is related to one of the most important myomiRs (also identified in our study), miR-133. Ray et al. [56] observed that miR-133 could reduce HuR mRNA and protein abundance. Also Legnini et al. [57] found a correlation between miR-133 and *elavl1*. The authors showed that HuR is repressed by miR-133 and that linc-MD1 alleviates this effect in early phases of differentiation in muscle. HuR may enhance the cellular growth in different kinds of tissues and also decreases apoptotic sensitivity to several chemical substances [58]. *rock1* seems to be an interesting gene identified in the DEG group. This gene was previously described as a negative regulator of myogenesis and a gene whose reduction promoted differentiation of both C2C12 and primary muscle cells [59].

### 4.3. Apoptosis, Oxidative Stress, and Immunity Related miRNAs and Genes

From a group of identified miRNAs, miR-92a, a part of the miR-17-92 cluster, which is a prototypical example of a polycistronic miRNA gene [60], seems to be interesting. This molecule is known to regulate cell viability and apoptosis, and is involved in antioxidant protection in different kinds of tissues [61]. miR-133a was reported to play similar role and its over-expression (also noticed in our experiment) protects cardiac myocytes from oxidative stress and inhibited cell apoptosis. Notably, miR-133a, miR-222, miR-502, miR-345, and miR-708 are related to apoptosis (activation and/or inhibition) in different kinds of tissue [62]. Attenuation of apoptosis in the course of the activation of satellite cells is essential for the proper course of regeneration. However, stimulation of apoptosis is necessary for removal of damaged cells at a later stage of regeneration.

Several identified miRNAs were previously described as those associated with processes that accompanied inflammation: blockade of miR-92a expression reduces inflammation. miR-188 seems to be one of the markers of inflammatory bowel disease [63]. Changes in miR-29 expression were observed during inflammation accompanying avian influenza infection. miR-208, which is closely related to cardiomyocyte, is also involved in cardiac fibrosis and inflammation by increasing myosin heavy chain (MHC) expression [64], and miR-222 (together with miR-10) is known to modulate endothelial inflammation [65].

Among a large group of identified genes, several were previously described as being involved in apoptosis. Some of them are known to have pro- and some anti-apoptotic properties. However, only a small number were related to apoptosis in muscle tissue. *Igf2,* which is an anti-apoptotic factor in muscle [66,67], was up-regulated in an experimental group. We observed a similar situation with *igf1r*; overexpression of this gene prevents apoptosis in different tissues. *rock1* is related to apoptosis in muscle; it induces multiple aspects of apoptosis including contractile force generation, membrane blebbing, and apoptotic-body formation [68].

Most studies about gamma-oryzanol described its strong antioxidant effect. In our study, we did not observe this effect on the scale that has been described so far. However, in an experimental group pre-treated with GO, we identified a group of miRNAs and genes related to oxidative stress in various tissue. Takahashi et al. [69] showed that *igf1r* activation prevents hydrogen peroxide-induced oxidative stress and apoptosis. Over-expression of *igf2* (also observed in our study) is strongly related to reducing free radical generation. *scd*, which is related to lipid enzymes activity and oxidative stress activation, was down-regulated in an experimental group. *setd6*, which was described as a negative regulator of oxidative stress, was down-regulated.

Over- and down-expression of selected miRNAs and genes in the GO pre-incubated group treated with hydrogen peroxide may suggest that these genes and miRNAs mediate the anti-apoptotic action of gamma-oryzanol and modulate this process that accompanies satellite cell injury induced by hydrogen peroxide.

### 4.4. Target Genes and Their Relation with Identified miRNAs and Selected Pathways

A large group of identified genes, which were described above, form relationships with several miRNAs. Based on the Target Scan analysis, we identified 22 potential differentially expressed target genes for the 17 identified miRNAs. Identified DET, together with related miRNAs, are involved in processes and several pathways known to be related in different processes, which accompany muscle tissue physiology and pathology. The identified pathways are presented in Table 4.

### 4.5. Insulin/Insulin-Like Growth Factor, (IGF) Pathway-Mitogen Activated Protein Kinase Kinase/MAP Kinase Cascade, AMPK Signalling Pathway and Gamma-Oryzanol Related Muscle Growth-miRNAs and Genes

**Figure 6:**
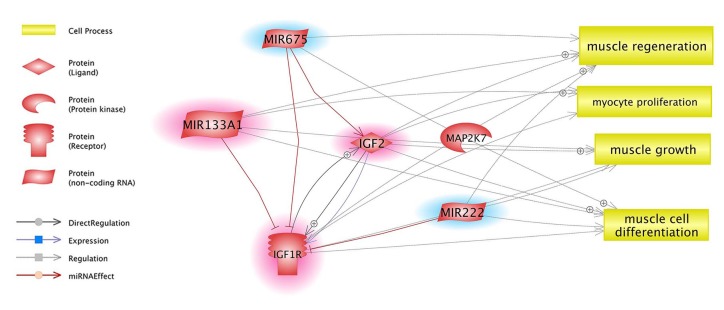
Relationships between DET and miRNAs, based on Pathway Studio Web Mammal. Genes are marked with red clouds and blue clouds for up- and down-regulation, respectively.

The insulin/IGF pathway-mitogen activated protein kinase kinase/MAP kinase (MAPK) cascade is one of the most interesting pathways related to muscles involving the potential activity of gamma-oryzanol in this tissue. Here, we identified three genes, *igfr1, igf2,* and *map2k7*, which were present in microarray analysis. Two of them were chosen as target genes for identified miRNAs. The MAPK cascade is closely related to muscle growth [69]. A preliminary study showed that gamma-oryzanol increases muscle growth, sports performance [70], and improved muscle strength [10]. In our study, we observed over-expression of all three aforementioned genes related to muscle growth and insulin/IGF pathway-mitogen activated protein kinase. These three genes are potential target genes for identified miRNAs—miR-675, miR-133a, and miR-222—which are known to also be involved in muscle growth and regeneration and muscle cell proliferation and differentiation (Figure 6).

Other pathway that is related to the genes and miRNAs identified in our experiment is the AMPK signaling pathway (also identified by DAVID) (Table 5), known to have critical roles in regulating growth and reprogramming metabolism. This pathway has recently been connected to cellular processes such as autophagy and cell polarity [71]. AMPK signaling has been thoroughly investigated in muscle tissue during various intensity exercises and different accompanying processes: cell/tissue adaptive response [72], oxidation in muscle [73], reactive oxygen species [74], contractile activity, and energy metabolism alterations [75].

Taken together, our study suggests that gamma-oryzanol may affect miRNAs and genes in injured differentiating equine satellite cells in multiple ways.

Several DET were related to various processes in different tissues (also muscle), which were previously described as being modulated by gamma-oryzanol. Their expression corresponds negatively with miRNAs expression, for which they were identified as target genes (Table 5).

### 4.6. Gamma-Oryzanol May Affect Metabolism-Related Genes and miRNAs

**Figure 7:**
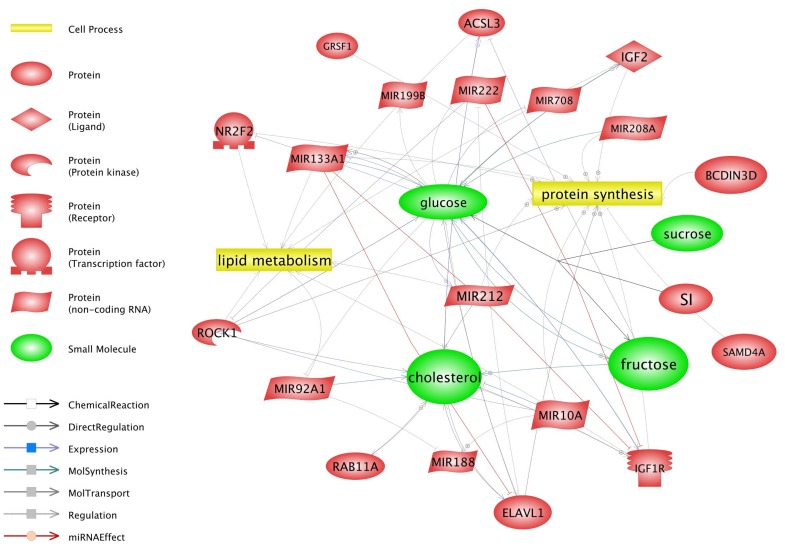
Relationships between differentially expressed genes, selected miRNAs and metabolic processes.

Gamma-oryzanol is known to affect lipid and carbohydrate metabolism [76,77]. Previous studies have shown the direct action of gamma-oryzanol on the expression of genes that encode proteins related to adiposity, inflammatory responses, and metabolic syndrome. In our study, we observed that, in a group that was pre-treated with GO, several miRNAs and genes related to the aforementioned processes had lower or higher expression levels compared with the control group (Figure 7). Among a large number of DEG associated with metabolic process, 50 were involved in primary metabolic process such as nucleobase-containing compound metabolic process, lipid metabolic process, cellular amino acid metabolic process, protein metabolic process, tricarboxylic acid cycle, and the carbohydrate metabolic process.

**Figure 8:**
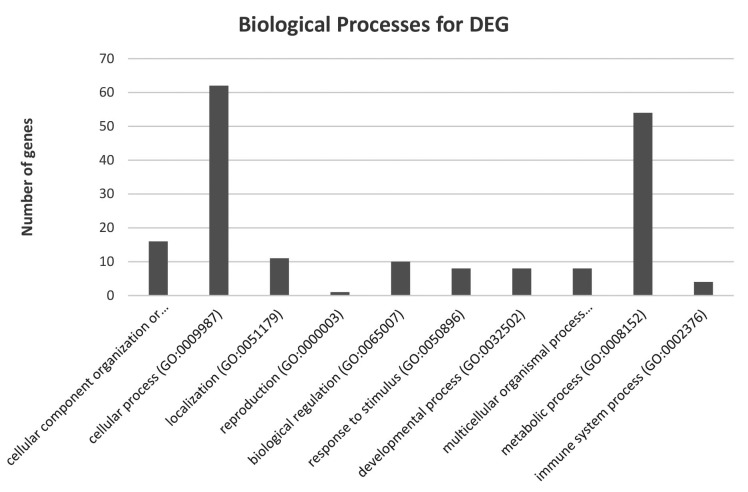
Number of DEG assigned to biological processes.

Figure 8 shows DEG related to different biological process. Table 6 provides the number of DEG related only to different primary metabolic process. Among the group of 10 identified processes, the second in terms of the number of genes is metabolic process. Based on the Pathway Studio Web Mammal analysis, we observed that miRNAs identified in an experimental group are also related to primary metabolic process and substances known to be associated with GO activity (Figure 7). Three of them, miR-212, miR-92a, and miR-133, are known to affect lipid metabolism. Guo et al. [78] suspected that miR-212 might be involved in lipid metabolism in mice. This miRNA promotes lipid accumulation and attenuates cholesterol efflux [79]. miR-10a is related to the cholesterol pathways. Jentzsch et al. [80] showed that this miRNA regulates cell size (hypertrophy) and increases protein synthesis in primary cardiomyocytes. Other interesting miRNA include miR-92a, which was shown to affect cholesterol metabolism and decrease liver and plasma cholesterol levels by modulating lipid-related genes in hamsters [81]. This miRNA is also related to the biosynthesis of insulin in rats [82]. Five identified miRNAs were previously described as those associated with glucose metabolism. MiR-133a that was reduced globally in mice improved insulin sensitivity and glucose metabolism. Huynh et al. [83] observed that concentration of glucose is closely related to miR-133a expression and described its potential role in cardiomyocytes hypertrophy. Crawford at al. [84] and Rodriguez-Comas et al. [85] also noticed a similar relation; however, they noticed the relation between the high level of glucose with the downregulation of miR-222 and miR-708. These findings, together with our observation, suggest that GO may affect the aforementioned processes. However, more data and several special tests are needed to better understand the direct effect of GO on ESC and its metabolism.

## 5. Conclusions

**Figure 9:**
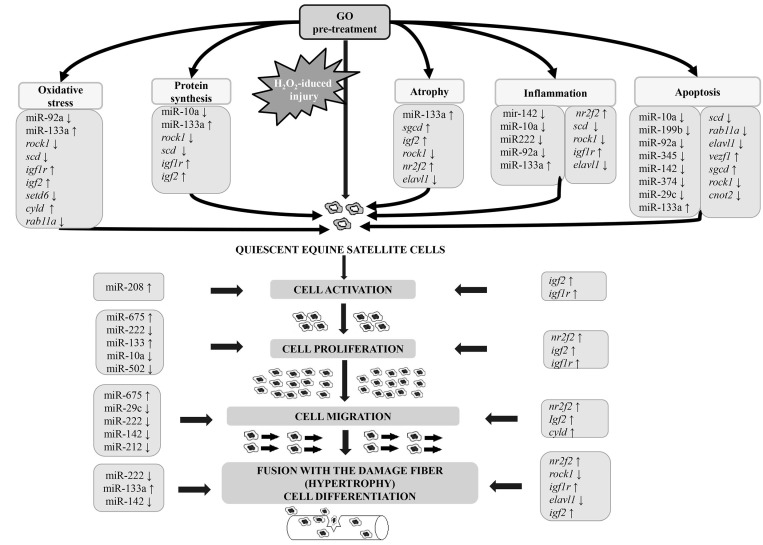
Potential role of GO-induced miRNAs and selected target genes in muscle regeneration process.

We demonstrated for the first time that GO-treated equine satellite cells exposed to H_2_O_2_ demonstrate a modulated expression of 17 miRNAs and 202 genes, which could affect the abovementioned processes. We found DET for identified GO-modulated miRNAs that are related to key processes in muscle physiology and pathology. We also identified signaling pathways related to muscle physiology and metabolism, providing new knowledge about the potential activity of gamma-oryzanol. The results related to TAC and lipid peroxidation showed that GO is an antioxidant and may protect ESC from free radicals activity associated with hydrogen peroxide treatment. These findings suggest gamma-oryzanol may be a potential myo-protectant that could prevent oxidative stress-induced physiological and pathological processes related to effort, some muscle diseases, and muscle tissue aging in horses (Figure 9). Using GO before hydrogen peroxide treatment showed that it may effectively protect against damage associated with free radical activity. However, more detailed research is needed, especially to check whether and how GO affects apoptosis and muscle regeneration.

Gamma-oryzanol is one of the most popular oral supplements used in performance equine nutrition as a clear substance or as in rice oil or rice bran. Based on previous studies suggesting that gamma-oryzanol can have effects similar to anabolic substances, gamma-oryzanol was placed on the list of banned substances by the Federation Equestre Internationale (FEI). However, it is often recommended as a measure aimed at restoring muscle mass in horses following trauma or a period of intense training (in the middle of competitions or racing season).

Our study provides new insight and creates new direction for the use of this additive. Potential anti-apoptotic activity may encourage us to use this substance in older horses in which muscle cell apoptosis can affect the proper function of musculoskeletal system, and in horses with different kinds of muscle diseases where the muscular cells are damaged.

## Figures and Tables

**Table 1:** Real-time qPCR primers

**(A)**					
**No.**	**Primer name**	**Target sequence**		**Amplification time and temperature**	
1	miR-133a	UUUGGUCCCCUUCAACCAGCUG		10 s/95 °C and 60 s/60 °C	
2	miR-345	GCUGACUCCUAGUCCAGUGCUC		10 s/95 °C and 60 s/60 °C	
3	miR-675	UGGUGCGGAGAGGGCCCACAGUG		10 s/95 °C and 60 s/60 °C	
**(B)**					
**No.**	**Primer name**	**Forward sequence**	**Reverse Sequence**	**Annealing time and temp.**	**Product length**
1	*nr2f2*	CCACCTCCTGCAGAACAAAAG	GGGTCTTGGTAAAGGCTCATT	15 s/60 °C	326
2	*gtpbp1*	CTCCAGACCACCAACAACTC	CCCCTGGGACTTCACCTTAT	15 s/62 °C	261
3	*nr2f2*	TCCTCCTCAGTCATAGAGCAAT	CTCTGTTTCACTCCCCTTTCTT	15 s/60 °C	340
4	*dtd1*	AGAGTTGGAGGAGAGCAGAT	TGGAGGGTAAACTGGCTGAT	15 s/60 °C	209
5	*btf3*	GCTCGCAGAAAGAAGAAGGT	GGTGCTTTTCCATCCACAGA	15 s/60 °C	329
6	*cs*	CGAGGCTACAGTATCCCTGA	CTTTCACTGTTGAGGGCTGT	15 s/60 °C	257
7	*gapdh*	GTTTGTGATGGGCGTGAACC	GTCTTCTGGGTGGCAGTGAT	15 s/60 °C	198

Note: (A) indiates miRNA; (B) indicates mRNA.

**Table 2:** MiRNAs differentially expressed in GO-incubated equine satellite cells exposed to H_2_O_2,_ compared to control.

No.	miRNA Name	FC (GO vs. ctrl)	Log FC (GO vs. ctrl)	Regulation (GO vs. ctrl)	miRbase Accession No
1	eca-miR-188-5p	2.18	1.13	up	MIMAT0013198
2	eca-miR-212	1.68	0.75	up	MIMAT0013030
3	eca-miR-685_v14.0	1.61	0.68	up	MIMAT0012904
4	eca-miR-133a	1.33	0.41	up	MIMAT0012997
5	eca-miR-208b	1.30	0.38	up	MIMAT0012900
6	eca-miR-675	−46.67	−5.54	down	MIMAT0013053
7	eca-miR-10a	−43.24	−5.43	down	MIMAT0013019
8	eca-miR-502-5p	−41.35	−5.37	down	MIMAT0013225
9	eca-miR-708	−40.90	−5.35	down	MIMAT0012993
10	eca-miR-345-5p	−33.19	−5.05	down	MIMAT0013138
11	eca-miR-142-3p	−28.34	−4.82	down	MIMAT0013023
12	eca-miR-324-3p	−1.75	−0.81	down	MIMAT0013034
13	eca-miR-199b-5p	−1.65	−0.72	down	MIMAT0013780
14	eca-miR-92a	−1.47	−0.56	down	MIMAT0013089
15	eca-miR-222	−1.45	−0.53	down	MIMAT0013204
16	eca-miR-874	−1.42	−0.50	down	MIMAT0013069
17	eca-miR-29c	−1.31	−0.39	down	MIMAT0012964

FDR ≤ 0.05, FC ≥ 1.3, *n* = 8, FC-Fold Change, FDR-False Discovery Rate.

**Table 3:** Real-Time qPCR validation of differentially expressed genes and miRNAs.

No.	GO vs. Control	Fold Change	*p*-Value
1	*nr2f2*	11.78	0.003
2	*btf3*	−6.37	0.005
3	*dtd1*	−6.32	0.007
4	*gtpbp1*	120.89	0.008
5	*otud4*	2.78	0.020
6	miR-133a	1.71	0.023
7	miR-345	−5.33	0.001
8	miR-675	1.39	0.038

**Table 4:** Identified signaling pathways and related DET.

Pathway	Genes	*p*-Value	FDR
AMP-activated protein kinase (AMPK) signaling pathway	*igf1r, scd, elavl1*	1.00 × 10^−2^	9.50
Proteoglycan in cancer	*igf1r, rock1, igf2*	2.80 × 10^−2^	24.0
Endocytosis	*igf1r, smap1, rab11a*	4.50 × 10^−2^	36.0
Fatty acid metabolism Peroxisome proliferator-activated receptor	*scd, acsl3*	6.30 × 10^−2^	47.0
(PPAR) signaling pathway	*scd, acsl3*	8.70 × 10^−2^	58.0

**Table 5:** DET and related processes together with corresponding miRNAs.

No.	Target Gene Symbol	Gene Description	Biological Processes	Fold Change (Microarray)	Related Identified miRNAs
1.	*acsl3*	Acyl-CoA Synthetase Long-Chain Family Member 3	Lipid biosynthesis and fatty acid degradation; Anabolic role in energy metabolism; Cell proliferation; Cell differentiation	−1.13	miR-142↓ miR-222↓
2.	*adamts3*	ADAM metallopeptidase with thrombospondin type 1 motif	Proteolysis; Protein processing; Inflammation	1.18	**miR-142↓**
3.	*bcdin3d*	BCDIN3 domain containing RNA Methyltransferase	Methylation; miRNA metabolic process	−1.13	miR-29↓ miR-502↓
4.	*cnot2*	CCR4-NOT transcription complex, subunit 2	Inflammation; Cytoplasmic deadenylation; DNA damage response; Regulation of stem cell population maintenance	−1.12	**miR-212↑** miR-222↓
5.	*cyld*	Cylindromatosis (Turban Tumor Syndrome)	Cell survival, proliferation, and differentiation; Innate immunity; Negative regulation of nf-kappa B import into nucleus	1.35	miR-133↑ miR-212↑
6.	*elavl1*	ELAV (embryonic lethal, abnormal vision, Drosophila)-like 1	Embryonic cell differentiation; Cytokine-induced cachexia; Cell growth and proliferation; Regulation of translation; Regulation of stem cell population maintenance	−1.23	**miR-133a↑ miR-208↑** miR-222↓ **miR-212↑**
7.	*grsf1*	G-rich RNA sequence binding factor 1	RNA processing; Cell proliferation; Protein synthesis: Apoptosis	−1.23	**miR-212↑** miR-199↓ **miR-208↑**
8.	*igf1r*	Insulin like growth factor 1 receptor	Apoptosis; Cell growth, differentiation and survival control; Immune response; Regulation of MAPK cascade; Oxidative stress	1.28	miR-133↑ **miR-502↓** **miR-675↓** miR-222↓
9.	*igf2*	Insulin-like growth factor 2	Growth factor activity; Striated muscle cell differentiation; Positive regulation of glycogen Biosynthetic process; Satellite cell proliferation	1.52	miR-133↑
10.	*mcmbp*	Minichromosome Maintenance Complex Binding Protein	A key regulator of pre-replication complex	−1.21	**miR-212↑** miR-142↓
11.	*nr2f2*	Nuclear receptor subfamily 2, group F, member 2	Muscle organ development: Cell migration; Cell proliferation; Inflammation	1.11	miR-212↑
12.	*rab11a*	RAB11A A member RAS oncogene family	Metabolic process; Inflammation; Marker for myopathies	−1.17	miR-142↓
13.	*rbpms*	RNA Binding Protein with Multiple Splicing	Response to oxidative stress; Heart, and gastrointestinal smooth Muscle development	−1.24	miR-199↓
14.	*rock1*	Rho-associated, coiled-coil containing protein kinase 1	Regulation of smooth muscle contraction; Regulation of actin filament-based process; Apoptotic process; Loss of muscle protein	−1.21	**miR-212↑**
15.	*samd4a*	Sterile Alpha Motif Domain Containing 4A	Cell junction; Modulates the activities of the mechanistic target of rapamycin complex 1; Regulates muscle/fat volume; Highly muscle-specific gene; (potential marker in dmd)	1.23	**miR-222↓**
16.	*scd*	Stearoyl-CoA Desaturase	Fatty acid biosynthesis; Biosynthesis of membrane phospholipids, cholesterol esters, and triglycerides; Cholesterol esterification; Muscle metabolism	−1.54	miR-199↓
17.	*serf2*	Small EDRK-Rich Factor 2	Marker of spinal and muscular atrophy; Muscle cachexia, muscle loss; Cell proliferation; Oxidative stress; Apoptosis	1.12	**miR-199↓**
18.	*setd6*	SET Domain Containing 6	Protein binding; Cell differentiation; Apoptosis	1.21	**miR-199↓** miR-133↑
19.	*sgcd*	Sarcoglycan, delta (35kDa dystrophin-associated glycoprotein)	Muscle organ/cell development; Cardiac muscle tissue development; Muscle dystrophy; Cell apoptosis	1.44	**miR-142↓**
20.	*smap1*	Small ArfGAP 1	Endocytosis; Cell differentiation; Apoptosis; Cell migration; Cell proliferation	−1.10	miR-10a↓
21.	*vezf1*	Vascular Endothelial Zinc Finger 1	Normal and abnormal cellular Proliferation and differentiation; DNA methylation	1.34	**miR-222↓ miR-142↓**
22.	*zswim6*	Zinc Finger SWIM-Type Containing 6	Metal ion binding; Dysostosis	1.20	miR-208↑

miRNAs in bold have the opposite expression change to corresponding DET. The arrows indicate the direction of expression change: ↓ and ↑ for down- and up-regulation, respectively.

**Table 6:** Primary metabolism processes and DEG.

No.	Process	Number of Genes	Genes
1	Nucleobase-containing compound metabolic process (GO:0006139)	21	*grsf1, atp6v0b, dis3, mcmbp, supt5h, nap1l1, mfn1, rab11a, rbpms, cwc22, snrpb2, samd4a, dtd1, ppil4, arhgap27, bcdin3d, strbp, ring1, nme2, sart1, dhx29*
2	Carbohydrates metabolic process (GO:0005975)	3	*sord, reep3, cs*
3	Cellular amino acid metabolic process (GO: 0006520)	4	*psph, cs, fahd2a, apip*
4	Lipid metabolic process (GO:0006629)	3	*nr2f2, acsl3, hadhb*
5	Protein metabolic process (GO:0019538)	20	*setd6, rwdd1, icmt, pigb, ilkap, mrps17, psmb4, gphn, spcs1, fkbp1a, dtd1, psmd14, ppil4, cyld, cnot2, adamts3, rpl29, pigg, eif3m, scara3*
6	Tricarboxylic acid cycle (GO:0006099)	1	*cs*

## Supplementary Materials

The following are available online at http://www.mdpi.com/2072-6643/10/12/1871/s1,Table S1: Genes differentially expressed in GO-incubated equine satellite cells exposed to H_2_O_2_ compared to control. FDR ≤ 0.05, FC ≥ 1.0, *n* = 4. Table S2: Target genes for all identified miRNAs.

## Abbreviations

AB	Antibiotic
CTRL	control condition
DEG	differentially expressed genes
DET	differentially expressed target genes
DM	differentiation medium
DMSO	dimethylsulfoxide
ESC	equine satellite cells
FBS	fetal bovine serum
FEI	Federation Equestre Internationale
GE	gene expression
GM	growth medium
GO	gamma-oryzanol
HS	horse serum
IB	incubation buffer
PBS	phosphate-buffered saline
ROS	reactive oxygen species
